# Simulations tackle abrupt massive migrations of energetic beam ions in a tokamak plasma

**DOI:** 10.1038/s41467-018-05779-0

**Published:** 2018-08-16

**Authors:** Andreas Bierwage, Kouji Shinohara, Yasushi Todo, Nobuyuki Aiba, Masao Ishikawa, Go Matsunaga, Manabu Takechi, Masatoshi Yagi

**Affiliations:** 1 Rokkasho Fusion Institute, National Institutes for Quantum and Radiological Science and Technology, 2-166 Oaza-Obuchi-Aza-Omotedate, Rokkasho, Kamikita, Aomori 039-3212 Japan; 2 Naka Fusion Institute, National Institutes for Quantum and Radiological Science and Technology, 801-1 Mukoyama, Naka, Ibaraki, 311-0193 Japan; 3 National Institute for Fusion Science, National Institutes of Natural Sciences, 322-6 Oroshi-cho, Toki, Gifu, 509-5292 Japan

## Abstract

In the late 1990s, fusion scientists at the Japanese tokamak JT-60U discovered abrupt large-amplitude events during beam-driven deuterium plasma experiments. A large spike in the magnetic fluctuation signal followed by a drop in the neutron emission rate indicates that energetic ions abruptly migrate out of the plasma core during an intense burst of Alfvén waves that lasts only 0.3 ms. With continued beam injection, the energetic ion population recovers until the next event occurs 40–60 ms later. Here we present results from simulations that successfully reproduce multiple migration cycles and report numerical and experimental evidence for the multi-mode nature of these intermittent phenomena. Moreover, we elucidate the role of collisional slow-down and show that the large-amplitude Alfvénic fluctuations can drive magnetic reconnection and induce macroscopic magnetic islands. In this way, our simulations allow us to gradually unravel the underlying physical processes and develop predictive capabilities.

## Abstract (web summary)

Understanding the occurrence of sudden changes in plasma parameters is important for the operation of magnetically confined fusion devices. Here the authors use simulation to shed light on the formation of abrupt large-amplitude events and the associated redistribution of energetic ions in a tokamak.

## Introduction

In the 2030s, large-scale fusion experiments with magnetically confined deuterium-tritium plasmas will be performed at the ITER^1^ tokamak, which is currently under construction in France. These experiments will test the idea of a self-sustained fusion plasma, where the energy deposited by fusion-born 3.5 MeV alpha particles eliminates the need for external heating. In order to ignite the plasma, however, a large amount of external heating will still be required, including a set of powerful negative-ion-based neutral beams (N-NB)^2^. Until now, the first and only tokamak equipped with such a system was JT-60U, which operated during 1985–2008 in Naka-city some 100 km north of Tokyo/Japan. The two N-NB lines in JT-60U were able to accelerate deuterium to energies as high as 400 keV at a combined power of up to 5 MW. Its successor JT-60SA^3^, which is scheduled to begin operation in 2020, will be equipped with an upgraded N-NB system designed to achieve 500 keV acceleration at 10 MW.

**Fig. 1:**
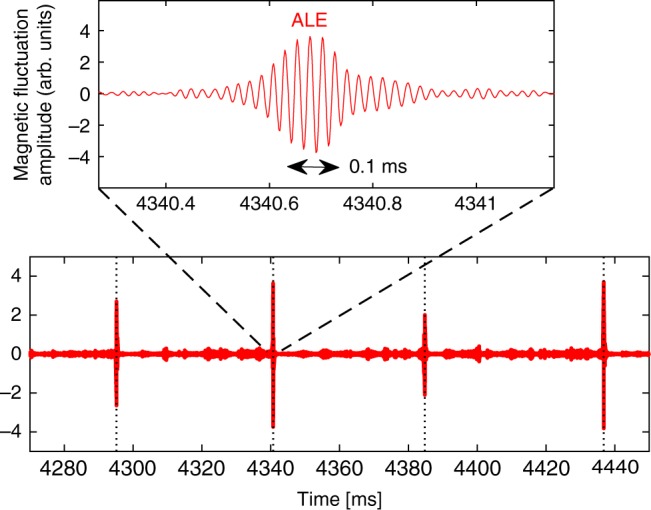
Abrupt large-amplitude events in the JT-60U tokamak. This time trace of the magnetic fluctuation amplitude in arbitrary units (arb. units) shows a sequence of four abrupt large-amplitude events (ALE) in JT-60U shot number E048424, which are visible here as large spikes that occur at intervals of 40–60 ms and last <0.3 ms each. The raw signal was measured with a sampling rate of 500 kHz using Mirnov pick-up coils that were located outside the plasma near the wall. Before plotting, the electric signal was time-integrated and band-pass filtered using a zero-phase forward and reverse digital Butterworth filter for the frequency range 30–70 kHz. An enlarged view of the second ALE is shown at the top, where one can see the rapid growth and decay of the Alfvénic oscillations with frequencies around 40–50 kHz. The time is measured in milliseconds (ms) relative to the start of the plasma discharge. The double arrow indicates a time interval of 0.1 ms

The N-NB-driven plasmas in JT-60U were found to exhibit short but intense perturbations dubbed abrupt large-amplitude events (ALE)^4^. During an ALE, the magnetic fluctuations can reach amplitudes 10 times larger than what is seen between these events. Figure 1 shows a typical example. Following the first observations of ALEs in the late 1990s^5^, intensive studies during subsequent experimental campaigns revealed that ALEs are usually accompanied by a massive migration of energetic deuterons from the plasma core into the periphery^6,7^. Since both JT-60SA and ITER will heavily rely on N-NB drive, it is important to understand ALE-like relaxation events and develop the ability to predict their occurrence in future experiments.

**Fig. 2:**
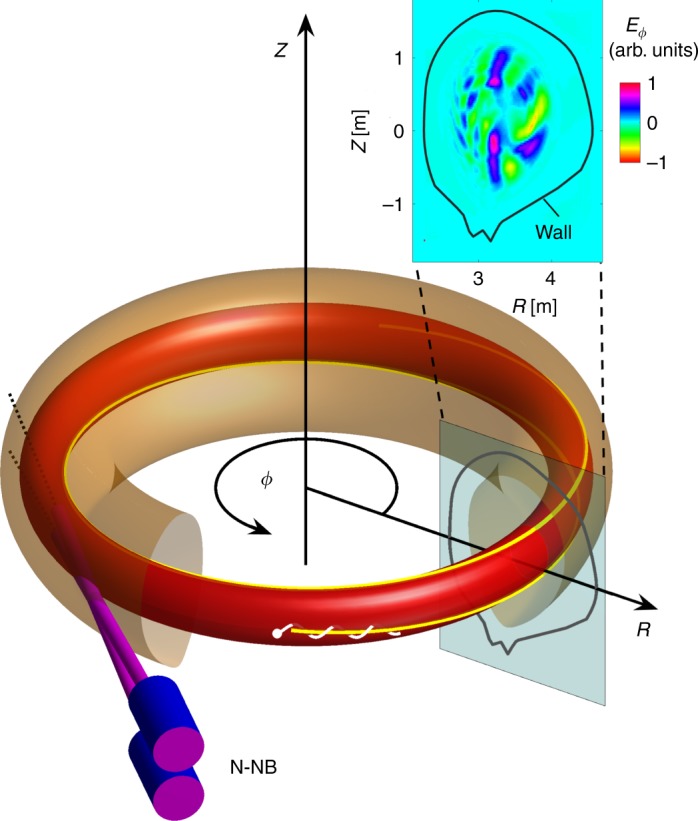
Geometry of the beam-plasma system. A pair of negative-ion-based neutral beams (N-NB, bottom left) injects neutralised deuterium tangentially into the JT-60U plasma torus, whose boundary is shown in light brown. In the absence of plasma fluctuations, the ionised 400 keV deuterons (white) gyrate around guiding centre orbits (yellow) that lie on toroidal surfaces such as the one shown in red. Collective resonant interactions between the energetic beam ions and shear Alfvén waves give rise to global fluctuations such as the example shown at the top right, where we have plotted the spatial structure of the toroidal component of the electric field *E*_*ϕ*_ in the poloidal (*R*,*Z*) plane at a certain toroidal angle *ϕ*, where *R* is the radial distance from the torus centre and *Z* the vertical coordinate. This snapshot of the fluctuating field *E*_*ϕ*_, which is shown here in arbitrary units (arb. units), was obtained with one of our simulations that are reported in this article. The blue and green regions represent **E** × **B** drift vortices with opposite orientations

The current theoretical understanding is as follows. Since N-NBs are injected tangentially into the plasma torus, as illustrated in Fig. 2, the beam ions are deposited with a steep density gradient and in a narrow range of pitch angles. Moreover, the velocity of fully accelerated ions is near or beyond the plasma’s Alfvén velocity. Under such conditions, and because the beam ions gradually slow down due to collisions with electrons, many particles will have a chance to interact with shear Alfvén waves^8^ through efficient transit resonances. Such a resonance occurs whenever the guiding centre velocity of the particle matches the local phase velocity of an Alfvén wave. The combination of a steep beam ion density gradient and efficient resonant drive can cause the waves to form globally coherent structures that withstand phase mixing (continuum damping) and reach large amplitudes^9,10^. An example of such a wave field is also shown in Fig. 2.

This fundamental wave–particle interaction process can produce a large variety of phenomena in magnetised plasmas and has also analogies in other areas of physics^11,12^. The particular phenomenology depends on the magnetic geometry, plasma parameters and the distribution of energetic ions, so that numerical simulations are usually needed to determine the response of a particular system.

Previous numerical studies aiming for an explanation of ALEs used educated guesses for what the energetic ion distribution may look like shortly before an ALE^13–20^. The simulation codes used in those studies employed efficient hybrid models^21^, where the thermalized $\left( { \lesssim 10\,{\mathrm{keV}}} \right)$ bulk plasma is described as a magnetohydrodynamic (MHD) fluid and the gyroaveraged motion of energetic ions $\left( { \gg 10\,{\mathrm{keV}}} \right)$ is simulated using the particle-in-cell (PIC) method. These studies have given us valuable insights concerning the response of the plasma on relatively short time scales of 1 ms or less. With suitably chosen initial conditions, it has even been possible to reproduce the experimentally observed abrupt flattening of the energetic ion density profile^15–17^.

Until now, the spatial and velocity distribution of the N-NB ions has been computed using orbit-following Monte-Carlo (OFMC) simulations^22,23^, which did not account for any MHD activity. When the OFMC simulation result was used as an initial condition for the N-NB ion distribution in a hybrid simulation^17^, the MHD fluctuation grew excessively large. The initial energetic ion pressure had to be reduced artificially by about a factor 2 in order for the hybrid simulation to reproduce the relative drop in the energetic ion pressure that had been inferred from neutron measurements before and after an ALE in a JT-60U experiment^7^.

In order to eliminate the need to predefine or tune the energetic ion distribution and make progress toward actual predictions, we have extended the hybrid code MEGA^13,14,24^ with a realistic model for N-NB ion sources and collisions^25^. In principle, this enables us to simulate the accumulation and distribution of energetic ions inside the plasma in the presence of continuous MHD wave activity. However, this is still a computationally extremely expensive task. Fortunately, it is not always necessary to simulate the MHD waves continuously. For instance, if one is interested only in an estimate of the pressure field of the energetic ions in the steady state, where beam deposition and transport are more or less in balance, it can be sufficient to simulate MHD activity for only 1 ms out of every 5–10 ms period; that is, only 10–20% of the time^26^. During the intervals where the MHD solver is turned off, all field fluctuations are set to zero, so the energetic ion population evolves only under the influence of collisions, sources and sinks in the unperturbed magnetic field. Each time the MHD solver is turned on, the fluctuations develop from zero. The speed-up gained from interlacing periods with and without MHD in this way makes long-time simulations covering hundreds of milliseconds feasible on presently available supercomputers^25^.

We have previously validated and benchmarked this method for cases with benign shear Alfvén wave activity and weak transport of energetic ions^27,28^. Here we show that this method also allows us to simulate intermittent large-amplitude bursts of Alfvénic fluctuations; namely, the ALEs shown in Fig. 1, along with the associated abrupt massive migration of energetic ions. In addition to improving the accuracy of predictions for the energetic ion pressure profile and velocity distribution, the elevated degree of self-consistency in the simulations allows us to learn more about the physical mechanisms whose interplay culminates in ALEs. First insights are reported, in part with supporting experimental evidence.

## Results

### Numerical simulation of multiple ALE cycles

For the present long-time simulations, we have chosen two similar cases whose stability and short-time response has been thoroughly examined in previous works^17,25,28,29^, where we have also justified the simulation parameters, benchmarked our code and numerical scheme, and validated the simulation results against other experimental observations. A detailed description of our methods and a list of all parameters can also be found in those references.

Both simulation scenarios are modelled on the basis of JT-60U shot E039672 at time *t* = 4 s, where the plasma current is *I*_p_ = 0.47M*A* and the so-called safety factor *q* (magnetic field line pitch) rises monotonically from *q* ≈ 1.3 at the centre to *q* ≈ 4 near the edge. In the original case^17^, the strength of the toroidal magnetic field is *B*_0_ = 1.16T at the plasma centre (magnetic axis). The modified case^25,28,29^ has a 3% stronger magnetic field *B*_0_ = 1.20T and a correspondingly higher Alfvén velocity *v*_A0_ = *B*_0_/(*μ*_0_*M*_D_*N*_i_)^1/2^, where *μ*_0_ is the vacuum permeability, *M*_D_ the deuteron mass, and *N*_i_ the bulk ion number density. Moreover, the injection energy $W_0{\mathrm{ = }}M_{\mathrm{D}}v_0^2/2$ of the beam ions was increased in proportion to $v_{{\mathrm{A}}0}^2$ by 7%: from 400 keV in the original case to 427 keV in the modified case. This was done in order to fix the beam-to-Alfvén velocity ratio at *v*_0_/*v*_A0_ = 1.432, so that the injected particles are equally super-Alfvénic in both cases.

**Fig. 3:**
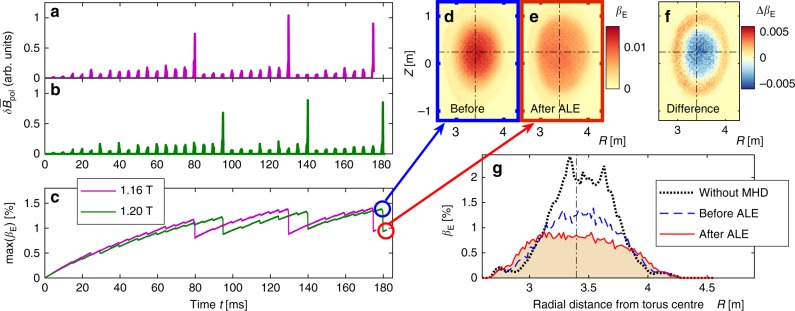
Long-time simulations of multiple ALE cycles. Two simulations were performed with different background magnetic field strengths, *B*_0_ = 1.16T and 1.20T, while fixing the beam-to-Alfvén velocity ratio *v*_0_/*v*_A0_ = 1.432. Panels **a** and **b** show the respective time traces of the magnetic fluctuation amplitude $\delta \bar B_{{\mathrm{pol}}}$. Panel **c** shows the evolution of the peak value of the beta parameter *β*_E_ for energetic ions with energies above 80 keV. During each ALE, max(*β*_E_) drops abruptly as beam ions migrate from the plasma core into the periphery. The extent of this spatial redistribution can be seen in panels **d**–**f**, where we compare snapshots of *β*_E_(*R*, *Z*) before and after the ALE at *t* = 180 ms in the *B*_0_ = 1.20T case. Panel **g** shows the radial profile *β*_E_(*R*) before and after the ALE at the height of the plasma midplane (*Z*_0_ = 0.2 m). For comparison, the dotted curve in **g** shows the overestimated *β*_E_(*R*) profile that one obtains when MHD activity is ignored. The dash-dotted lines in **d**–**g** indicate the position of the plasma centre; namely, the magnetic axis

The long-time simulation results are summarised in Fig. 3. At the beginning of each simulation, the plasma is in a stable MHD equilibrium and we initiate the continuous deposition of energetic deuterons via two N-NB lines with a combined power of 5 MW. The energetic beam ions gradually slow down, primarily due to collisional drag exerted by thermal electrons with temperature *T*_e_ ≈ 2 keV and number density *N*_e_ ≈ 2.2 × 10^19^ m^−3^. Note that, for these plasma parameters, it takes up to 0.5 s for a beam ion to thermalise; that is, to slow down to a few keV.

Figure 3a, b shows the time traces of the volume-averaged magnetic fluctuation amplitude $\delta \bar B_{{\mathrm{pol}}} \equiv \left[ {V^{ - 1}{\int} {\mathrm{d}}V\left( {\delta B_{{R}}^2 + \delta B_{{Z}}^2} \right)} \right]^{1/2}$, where *δB*_*R*_ and *δB*_*Z*_ are the fluctuating components of the magnetic field in the poloidal (*R*, *Z*) plane. During the first 80–95 ms, there are only weak fluctuations whose amplitudes increase gradually in time as an increasing number of beam ions is accumulated in the plasma. This gradual rise of low-amplitude fluctuations is abruptly terminated by a large spike, which occurs at 80 ms in the 1.16 T case and at 95 ms in the 1.20 T case. After that, the cycle of gradually increasing low-amplitude fluctuations followed by a large spike repeats at intervals of 40–50 ms.

The simulated evolution of the magnetic fluctuations in Fig. 3a, b closely resembles experimental observations of ALEs, such as those shown in Fig. 1 above. Moreover, the similarity of the results obtained in our two simulation scenarios demonstrates the reproducibility of the ALE simulations. At the same time, one can get an idea about the degree of sensitivity of the results for small changes in the parameter values.

A direct comparison of the fluctuation amplitudes in simulations and experiments is not possible because experimental measurements could only be performed outside the plasma at the wall, whereas measurements in the simulations must be made inside the plasma, away from the artificially fixed plasma boundary. For the sake of completeness, we note that the external magnetic fluctuation amplitudes measured by Mirnov coils are of the order $\delta B_{{\mathrm{exp}}}^{{\mathrm{external}}}\sim 10^{ - 4}B_0$ during an ALE and ~10^−5^*B*_0_ between ALEs. The internal magnetic fluctuations in the simulations are of the order $\delta B_{{\mathrm{sim}}}^{{\mathrm{internal}}}\sim 10^{ - 2}B_0$ during an ALE and ~10^−3^*B*_0_ between ALEs.

It is interesting to note that the 80–95 ms period that passes before the first ALE is significantly longer than a typical ALE interval of 40–60 ms. We assert that, when the first ALE occurs, the energetic ions have just barely filled the resonant regions of the velocity space, whereas all subsequent ALEs occur in the presence of a fully developed energetic ion tail. On the basis of this argument, we estimate that particles driving ALEs can be found within the energy range $100{\kern 1pt} \,{\mathrm{keV}} \lesssim W \lesssim W_0$, because only this range is fully populated by the time the second ALE occurs around 120–130 ms after the beginning of the simulation (see the velocity distributions in ref.^17^).

This observation justifies a posteriori our practice of discarding simulation particles that have slowed down below 80 keV. In fact, in the experiments, the ion population below 80 keV is strongly modified by other beams, which are used for plasma heating and diagnostics purposes and which are not included in our simulations.

### Massive migration of energetic beam ions

Each ALE causes an abrupt large change in the energetic ion distribution. This can be gleaned from the sawtooth-like pattern in the time traces of the peak value of the energetic ion beta parameter *β*_E_ in Fig. 3c and, in more detail, from the snapshots of its spatial distribution in Fig. 3d–g. The dimensionless beta parameter $\beta _{\mathrm{E}} = 2\mu _0P_{\mathrm{E}}/B_0^2$ measures the ratio of the kinetic pressure *P*_E_ of the energetic ions to the magnetic energy density $B_0^2/(2\mu _0)$. This quantity *β*_E_ (strictly speaking, its gradient) serves us as a simple measure for the strength with which the beam ions can destabilise shear Alfvén waves.

At the beginning of the simulation, the beam ion distribution is effectively monoenergetic, so that $\beta _{\mathrm{E}} \approx 2N_{\mathrm{E}}v_0^2/\left( {N_{\mathrm{i}}v_{{\mathrm{A}}0}^2} \right)$, where *N*_E_ is the number density and *v*_0_ the birth velocity of the beam ions. Both simulated cases have the same particle injection rate (and thus *N*_E_), the same bulk ion density *N*_i_ and the same velocity ratio *v*_0_/*v*_A0_. This explains why the two time traces of *β*_E_ in Fig. 3c are approximately equal during the first 10–20 ms of both simulations. Any deviation in the subsequent evolution of the two cases can be attributed to the randomness of the collisions and the small difference in the input parameters. In particular, the 3% difference in the background magnetic field strength *B*_0_ influences the spatial structure and frequency spectra of the MHD fluctuations, the energetic particle trajectories and their resonance conditions.

The snapshots in Fig. 3d–g show that a large portion of the energetic ion population migrates from the core plasma into the periphery. In the example shown, this massive migration causes the energetic ion pressure in the centre of the plasma to drop by about 30% in <0.3 ms, which corresponds to only about a dozen oscillation periods of the shear Alfvén wave field.

For comparison, the dotted curve in Fig. 3g shows the energetic ion beta profile that one obtains if MHD activity is entirely ignored, as in a conventional Monte-Carlo particle simulation. One can see that such a simulation largely overestimates the energetic ion pressure by at least a factor 2 in the present case. This kind of discrepancy has been a long-standing issue, which affects not only the prediction of energetic ion profiles, but also the accuracy of MHD equilibrium reconstruction, since the beam ions contribute to the current drive, heating and torque. Large discrepancies can occur even in cases where the energetic ion pressure saturates near a more or less steady state^30^. Comprehensive long-time hybrid simulations such as those reported here offer a way to improve the predictions for cases with relatively steady energetic ion pressure^26,27^, as well as abrupt relaxation events such as the present ALEs in JT-60U, albeit at the expense of consuming nearly 100 times larger computational resources than a Monte-Carlo simulation and even more compared to simulations using reduced (low-dimensional or ad hoc) transport models.

Having reproduced a sequence of ALEs numerically, our simulations can be used in the future as a tool for obtaining insights concerning when and why ALEs occur. Such information may help researchers to construct or improve reduced models, which are more efficient computationally and are urgently needed for the development of experimental scenarios and plasma control. Recent promising work on that front includes the use of stochastic annealing across overlapping resonances^31,32^, statistical kick models^33,34^, and the definition of critical gradients in the energetic ion distribution near the most important resonances^35^. For this purpose, one has to be able to follow the evolution of the energetic ion distribution and characterise the properties of the MHD fluctuations during an ALE. Our simulations can be used to make further progress in that direction by serving as a close-to-first-principle benchmark and by providing the necessary physical insights. Below are our first findings.

### Recovery of the pressure profile and velocity distribution

**Fig. 4:**
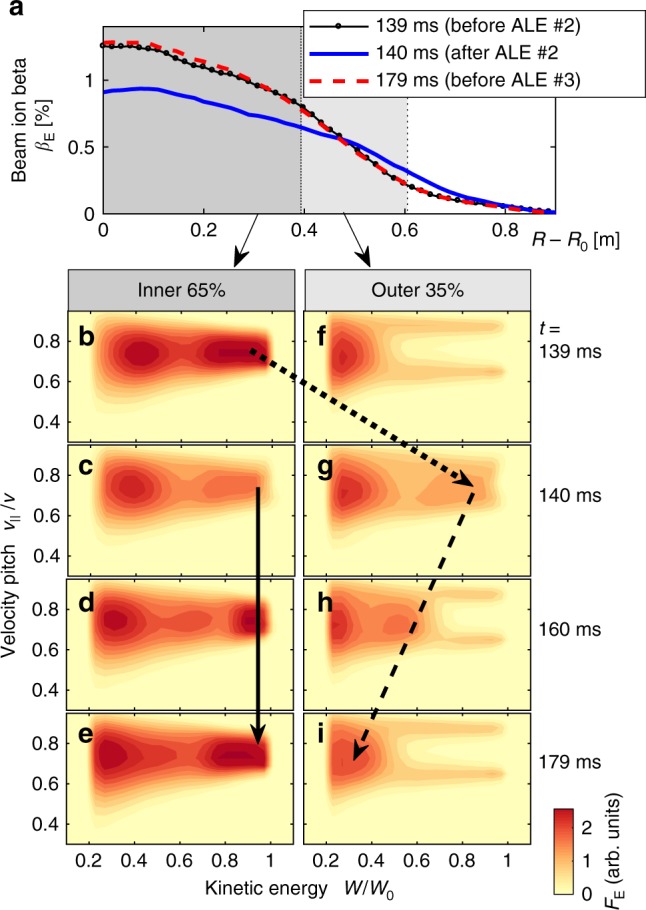
Evolution of the beam ion distribution. For the time interval between the second and third ALE in the *B*_0_ = 1.20T case, panel **a** shows the evolution of the energetic ion beta profile *β*_E_(*R* − *R*_0_) measured in the midplane (at *Z*_0_ = 0.2 m), where *R* − *R*_0_ is the radial distance from the plasma centre (at *R*_0_ = 3.4 m) towards the outboard side of the tokamak. Note that the energetic ion orbits extend beyond the plasma boundary (located at *R*−*R*_0_ = 0.6 m) into the surrounding vacuum (white area). The profiles in **a** were smoothed by averaging over 10 radial grid points, which are indicated as dots on the first profile (black). Panels **b**–**e** show the evolution of the velocity distribution $F_{\mathrm{E}}(W,v_{||}/v)$ in the core (inner 65% of the plasma), and panels **f**–**i** in the periphery (outer 35%). The kinetic energy *W* = *M*_D_*v*^2^/2 of the deuterons is normalised by their birth energy *W*_0_. The velocity pitch is measured by the ratio $v_{||}/v$, where $v_{||}$ is the velocity component along the magnetic field. The dotted arrow from **b** to **g** indicates the abrupt migration of energetic particles from the core plasma into the periphery during the interval 139 ms ≤ *t* ≤ 140 ms (ALE #2). The solid arrow from **c** to **e** indicates how the beams replenish the energetic ion population in the core until *t* = 179 ms (before ALE #3). Meanwhile, the number of energetic ions in the periphery decreases, because the migrated particles slow down via collisional drag as indicated by the dashed arrow from **g** to **i**

After each ALE, a peaked energetic ion pressure profile is restored under the influence of sources and collisions, in spite of continued low-amplitude MHD wave activity. In this restoration process, the pressure gradually increases in the centre, where the beams are injected. Meanwhile, the pressure in the periphery gradually decreases. This can be seen in Fig. 3g and is shown in more detail in Fig. 4a, where we plot *β*_E_(*R* − *R*_0_) on the outboard side (*R* ≥ *R*_0_) of the equatorial plane. Three snapshots are shown for the *B*_0_ = 1.20 T case: shortly before and after the 2nd ALE, and just before the 3rd ALE.

The above-mentioned peripheral pressure reduction may be important as it enhances the steepening of the gradient before each ALE. It is due to the higher collisionality in the cooler peripheral plasma, which causes the energetic particles that were expelled by the preceding ALE to slow down more rapidly than those that remained in the core. This process can be seen in the bottom part of Fig. 4, where we show a series of snapshots of the beam ion velocity distribution between the 2nd and 3rd ALE in the *B*_0_ = 1.20T case. The horizontal axis is the kinetic energy *W* = *m*_D_*v*^2^/2 normalised by the birth energy *W*_0_. The vertical axis is the velocity pitch $v_{||}/v$, where $v_{||}$ is the component along the magnetic field. One can see how the two beams are deposited in the core with similar pitches around $v_{||}/v \approx 0.75$. In the periphery, their respective pitches are approximately $v_{||}/v \approx 0.65$ and 0.9.

Fig. 4b–e shows the velocity distribution integrated over the inner 65% of the plasma radius, where most of the beam ions are deposited, so that a bump-on-tail distribution is formed. The situation for the outer 35% of the plasma is shown in Fig. 4f–i, where one can see how the energetic ion tail nearly disappears during the interval between the ALEs. While the gradient steepening may not appear to be dramatic in the velocity-averaged beta profile in Fig. 4a, the different evolution of the velocity distribution in the core (Fig. 4b–e) and in the peripheral plasma [Fig. 4f–i] implies that there is a substantial steepening in the pressure gradient associated with particles with energies above 200 keV (or *W*/*W*_0_ > 0.5).

Therefore, it is likely that reduced predictive models for ALEs must take into consideration how sources and collisions affect the gradients near the various resonances in different regions of velocity space. Before this can be done, it is necessary to have some knowledge about the spectrum of Alfvénic modes that are excited during ALEs. Below are our latest insights in this regard.

### Multi-mode nature of ALEs

In the present paper, we use the word mode in a loose manner, usually referring collectively to all fluctuations that have the same wavelength along the plasma torus. These can be isolated by Fourier-analysing the signals along the toroidal angle *ϕ* (see Fig. 2) using the basis functions sin(*nϕ*) and cos(*nϕ*), where the integer *n* is called toroidal mode number. The wavelength of the *n* = 1 harmonic equals the length of the torus, for *n* = 2 it is half the torus length, and so on. This decomposition is meaningful because of the toroidal symmetry of a tokamak plasma, which implies that Fourier harmonics with different values of *n* constitute independent modes that interact only nonlinearly. Of course, when separated in radial location and frequency, independent modes with the same value of *n* may also exist.

**Fig. 5:**
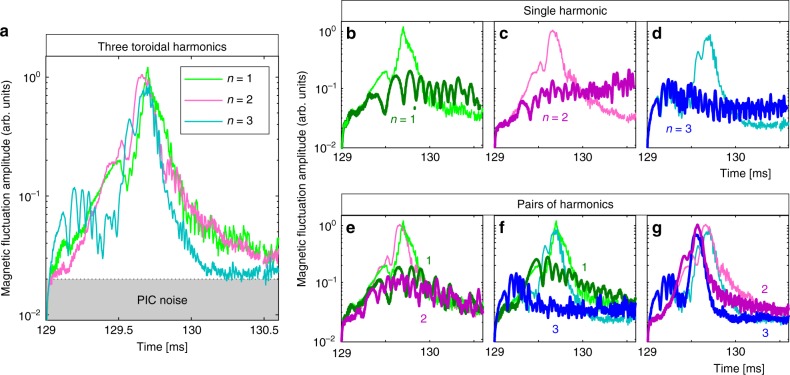
Numerical prediction of the multi-mode nature of ALEs. The time interval 129 ms ≤ *t* ≤ 131.6 ms around the second ALE in the *B*_0_ = 1.16 T case of Fig. 3 was simulated again without beam injection and without collisions, but with continuous MHD activity. The magnetic fluctuation signal $\delta \bar B_{{\mathrm{pol}}}$ was decomposed into Fourier harmonics with toroidal mode numbers *n* = 1,2,3, whose time traces are shown in panel **a**. This simulation was then repeated six more times, while keeping only one harmonic at a time or pairs of harmonics: **b** *n* = 1, **c** *n* = 2, **d** *n* = 3, **e** *n* = 1 and 2, **f** *n* = 1 and 3, **g** *n* = 2 and 3. For comparison, the respective time traces from **a** are plotted once more in **b**–**g** as light-coloured curves

The first attempts to explain ALEs considered only the *n* = 1 mode^15,16^, but more recent studies suggest that multiple modes may be involved^17–20^. In Fig. 5, we present results that provide direct evidence for the multi-mode nature of ALEs. In our simulations, the Fourier harmonics with *n* = 1, 2, 3 where directly driven by energetic ions. The *n* = 0 and *n* = 4 harmonics provide channels for nonlinear damping, and harmonics with *n* = 5 and larger were filtered out because they do not seem to be crucial here.

Since we need to look only at a short time window of about 1 ms duration, we have repeated the simulation of the ALE at 129 ms in the 1.16 T case without beams and without collisions. This gives us a well-defined initial condition and eliminates pseudo-random numbers from the simulation. For this simplified setup, Fig. 5a shows the time traces of the magnetic fluctuation amplitude for each of the three Fourier harmonics *n* = 1,2,3 that receive direct drive from energetic ions in this simulation. One can see that all three modes reach a large amplitude more or less simultaneously.

Let us now examine the question whether some kind of synergy between these modes is essential or not. Starting again from time *t* = 129m*s*, we have repeated this simulation while including only a single mode (Fig. 5b–d) or pairs of modes (Fig. 5e–g). One can see that the fluctuations remain weak in all three single-*n* simulations, and become stronger when pairs of modes are combined. In this particular case, the largest response—similar to that seen in the full simulation with all three harmonics—was obtained when combining *n* = 2 and *n* = 3.

Although the *n* = 1 mode was not essential for producing a large-amplitude response on the short time scale in the particular case shown in Fig. 5, that mode is always observed during the relatively quiet intervals between ALEs. Its frequency-chirping and bursty dynamics^13,15,16,20,25^ are likely to play a key role in producing the conditions under which an ALE can be triggered.

**Fig. 6:**
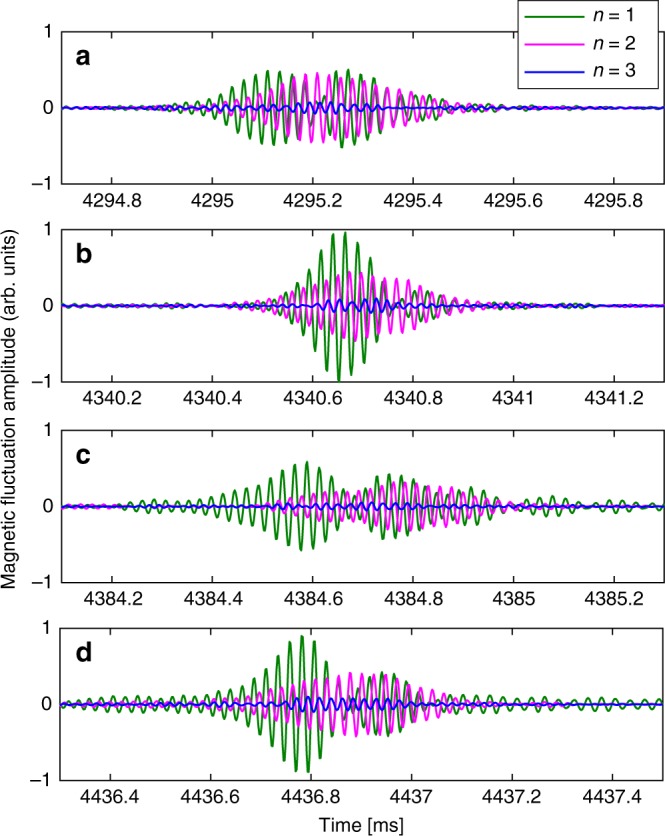
Experimental confirmation of the multi-mode nature of ALEs. Panels **a**–**d** show a 12 ms time window around one of the four ALEs that can be seen in Fig. 1. The magnetic fluctuation signal that is plotted here in arbitrary units (arb. units) was measured in JT-60U shot number E048424 using a toroidal array of saddle loop coils. The raw electric signal was sampled at 500 kHz, time-integrated, and band-pass filtered using a zero-phase forward and reverse digital Butterworth filter for the frequency range 30–70 kHz. The result was then decomposed into toroidal Fourier harmonics with mode numbers *n* = 1, 2, 3, whose time traces are plotted here in different colours. One can see that modes with *n* = 1 and *n* = 2 reach large amplitudes during each ALE. The measurement sensitivity is significantly lower for modes with *n* = 3 and higher; namely, shorter wavelengths

The simulation results in Fig. 5 predict that ALEs have a multi-mode nature and this finding motivated us to search the JT-60U database for experimental evidence that may confirm this prediction. One technical drawback is that tomographic measurements of the spatial structure of Alfvénic fluctuations using solid-state detectors could not be performed in JT-60U deuterium plasma experiments with strong N-NB drive, as the diagnostics were likely to be damaged by the high fluxes of gamma rays and neutrons. Therefore, only signals from external magnetic measurements are available. Using a system of so-called saddle loops and high-frequency data acquisition, it was possible to clearly distinguish Alfvénic harmonics with *n* = 1 and 2, and to some extent *n* = 3. No reliable measurements exist for *n* > 3. We have analysed all four ALEs seen in Fig. 1 and the results are summarised in Fig. 6.

The relative amplitude of the *n* = 3 harmonic in the experimental data in Fig. 6 is much weaker than in the simulation results in Fig. 5. As discussed in the Methods section below, this can be explained with the fact that the saddle loops are much less sensitive to signals with *n* > 2. Apart from this, the experimental results in Fig. 6 seem to confirm the multi-mode nature of ALEs predicted by theory and simulation. Moreover, one can readily see from Fig. 6 that each ALE looks different from the others, even in the same plasma discharge. This suggests that there are many different ways in which such events can be realised, which may explain the robustness with which ALEs are observed under a wide range of experimental conditions. This also implies that the simulations need not reproduce any particular pattern of multi-mode interactions.

### Nonlinearly driven magnetic reconnection

**Fig. 7:**
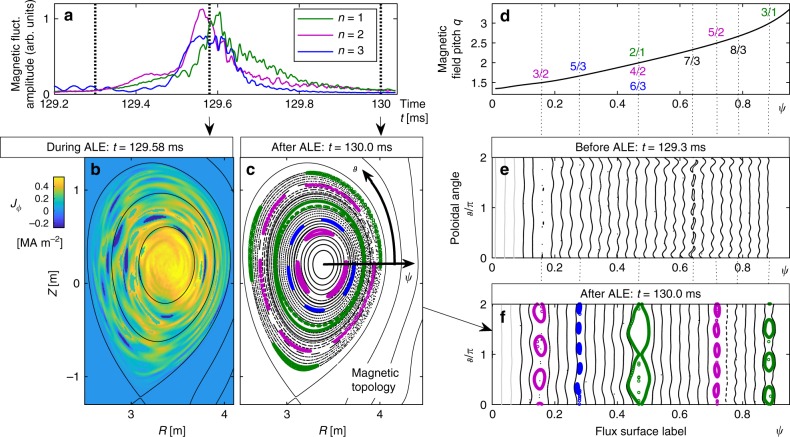
Magnetic reconnection during an ALE. For the second ALE in the *B*_0_ = 1.16T case, panel **a** shows the evolution of the magnetic fluctuations decomposed into toroidal harmonics *n* = 1,2,3. Panel **b** shows a vertical cross-section of the toroidal current density *J*_*ϕ*_(*R*, *Z*) at *ϕ* = 0 during the ALE, and **c** shows a Poincaré plot of the magnetic field topology after the ALE. Magnetic island chains are highlighted in colours corresponding to the dominant toroidal mode number *n*. The solid black lines in panels **b** and **c** indicate the unperturbed magnetic surfaces, which also correspond to contour lines of the magnetic flux function *ψ*. Using *ψ* as a radial-like coordinate and introducing a suitable angular coordinate *ϑ*, we obtain a polar set of coordinates as indicated by the arrows in panel **c**. The same Poincaré data as in **c** but mapped into the (*ψ*,*ϑ*) plane is shown in panel **f**, and the situation before the ALE can be seen in **e**. Here, the magnetic islands form at resonant magnetic surfaces *ψ*_*m*/*n*_ where *q*(*ψ*_*m*/*n*_) = *m*/*n* = 3/2, 5/3, 2/1, 5/2 and 3/1. Their locations can be inferred from panel **d**, where we show the mean profile of the magnetic field line pitch *q*(*ψ*), commonly known as the tokamak safety factor

It is known that large-amplitude MHD fluctuations cannot have pure interchange parity. In the nonlinear regime, there is always a certain amount of mixing with tearing parity components^36^. We suspect that this parity mixing may be enhanced by energetic beam ions, whose currents are highly anisotropic and radially shifted by magnetic drifts. In the presence of dissipation (when collision-dominated) or saturation effects (when collisionless), the tearing components of the resonantly driven modes lead to changes in the magnetic topology. The results summarised in Fig. 7 show that a significant amount of magnetic reconnection occurs during the ALEs in our resistive hybrid simulations.

Similarly to Fig. 5a, we restarted the simulation of the *B*_0_ = 1.16 T case from *t* = 129 ms with continuous MHD activity. Figure 7a shows the evolution of the *n* = 1, 2, 3 harmonics in that simulation. For *t* = 129.58 ms, where the ALE reaches its peak, Fig. 7b shows a snapshot of the toroidal current density field *J*_*ϕ*_(*R*,*Z*) at *ϕ* = 0. One can see that the large-amplitude magnetic fluctuations during the ALE come with substantial filamentary perturbations of the toroidal current density, which can exceed the local background current density. Although the ALE pulse lasts only about 100 *μ*s, the highly corrugated filamentary structure of *J*_*ϕ*_ suggests that some portion of the current may already undergo resistive dissipation and facilitate magnetic reconnection.

Figure 7c shows a Poincaré plot of the magnetic field topology after the ALE at *t* = 130.0m*s*. Four magnetic island chains are highlighted in colour, where each colour identifies the respective dominant toroidal mode number *n*. Figure 7f shows the same Poincaré plot in another projection. The horizontal axis is the flux surface label *ψ*, which is a radial-like coordinate with *ψ* = 0 in the centre (magnetic axis) and *ψ* = 1 at the plasma boundary. The vertical axis is the poloidal angle *ϑ*. These coordinate axes are indicated by arrows in Fig. 7c.

As expected, the island chains are located at resonant magnetic surfaces *ψ*_*m*/*n*_, where the magnetic field pitch (tokamak safety factor) has rational values *q*(*ψ*_*m*/*n*_) = *m*/*n* as indicated in Fig. 7d. For each island chain, the poloidal mode number *m* tells us how many times one island tube crosses the poloidal plane in Fig. 7c, f before closing upon itself. Note that the island chain at *ψ*_2/1_ ≈ 0.47, where *q* = 2/1 = 4/2 = 6/3, is dominated by the *n* = 1 mode and distorted by higher harmonics with *n* = 2 and 3. The magnetic islands were effectively absent before the ALE as one can see from the Poincaré plot for *t* = 129.3m*s* in Fig. 7e. At that time, there were only Alfvén waves, which appear as wiggles on the magnetic surfaces. Unperturbed magnetic surfaces appear as straight vertical lines.

At present, it is not known whether a significant amount of magnetic reconnection occurs during ALEs in real-world experiments, where electric resistivity is negligibly small, but in its place there are other nonideal effects, such as finite Larmor radii and electron inertia, which are not present in our simulations. In any case, the role of magnetic reconnection must be taken into account when analysing and interpreting the predictions of resistive hybrid simulations such as those presented here. It should be noted that the energetic ions are not trapped in magnetic islands but in drift orbit islands. These are influenced by the electric and magnetic drifts of the charged particles and have different existence conditions, spatial locations and widths than magnetic islands^37,38^.

The analysis of all these effects is underway with the aim of clarifying the ALE trigger mechanism. This is a difficult task due to the participation of multiple modes with different wavelengths and different frequencies, and because ALEs last only a dozen or so Alfvén oscillation cycles with rapidly increasing and decreasing amplitudes.

## Discussion

The results reported above show that, using presently available supercomputers, it is now possible to reproduce intermittent massive migrations of energetic N-NB ions during ALEs as observed in JT-60U experiments and gain insights concerning the underlying physical processes.

While previous estimates of the energetic ion pressure obtained from Monte-Carlo simulations had to be rescaled by a factor 1/2 in order to reproduce the observed amount of transport when plugged into a conventional hybrid simulation^17^, the comprehensive long-time simulations performed here eliminate the need for such tuning by integrating the realistic beam and collision model of the Monte-Carlo simulation into the hybrid code MEGA^25,26^. Absolute quantitative measurements of the energetic ion distribution in ALE experiments are not available, so we are not able to evaluate the accuracy of the predictions directly. However, the energetic ion beta values obtained in the present simulations (*β*_E_ ≈ 1%–1.5% at the plasma centre) imply that the total neutron emission rate of about (0.6–1.0) × 10^15 ^s^−1^ for the N-NB component is consistent with experimental measurements, as was shown in the summary figure of ref.^16^. Moreover, a recent validation study against a well-diagnosed DIII-D tokamak experiment^27^ showed that MEGA results were an order of magnitude more accurate than predictions based on collisional transport only. In that case, the remaining discrepancies fell within experimental error bars of about 20% in the plasma core. The improved predictions for the energetic ion distributions are an essential prerequisite for making reliable predictions for the current drive, torque and heating power that the energetic particle beams exert on the bulk plasma. These factors, in turn, influence many other self-organisation processes that occur in magnetically confined plasmas — from microturbulence to large-scale MHD instabilities.

Further work is required in order to fully unravel the mechanisms that trigger ALE-like relaxation events and to become able to construct computationally efficient yet reliable reduced models. The analysis of the simulated ALE dynamics is underway and first insights were reported here.

We have presented direct evidence, both numerical and experimental, that multiple Alfvén modes with toroidal mode numbers in the range *n* = 1, 2, 3 are present during an ALE, and that interactions between them are essential for producing a large-amplitude response. These interactions are most likely dominated by indirect couplings, where multiple modes (or wave packets) interact with a certain group of particles via intersecting resonances^17,19,39^ or via the propagation, coalescence and overlap of nonlinear phase space structures^40–47^. Untangling these interactions during a short event like an ALE is a difficult task and a subject of ongoing research.

We have shown that the steepening of the energetic ion pressure gradient during the intervals between successive ALEs is not caused by the continuous beam injection alone; it is also aided by the nonuniform collisionality, which causes energetic ions in the cooler peripheral plasma to slow down more rapidly than in the hotter core. Moreover, the collisional slow-down carries nonuniformities in the beam ion distribution from high to low energies, so that these nonuniformities come across various resonances in velocity space. This allows perturbations created by the beams or wave-particle interactions at higher energies to cause retarded responses when they reach resonances at lower energies. In principle, this gives rise to the possibility that an ALE is well-separated in time from the trigger event. Therefore, it is important to clarify the role of the experimentally observed low-amplitude bursts of chirping modes^25^ that occur between ALEs at intervals of about 5–10 ms. In the present simulations, these low-amplitude bursts are induced somewhat artificially as the MHD solver is turned on and off periodically. In a fully self-consistent simulation with continuous MHD activity, it is possible that MHD nonlinearities^48^ in combination with dissipation at small scales in phase space^49–52^ play a key role for facilitating the steepening of the pressure gradient between ALEs by suppressing or delaying instabilities before an ALE occurs.

Finally, we have shown that the energetic-ion-driven large Alfvénic oscillations during an ALE are associated with large filamentary perturbations of the plasma current, which give rise to magnetic reconnection in our resistive hybrid simulations. The resulting magnetic islands can have a much longer life time than the shear Alfvén waves and may play an important role for the confinement of both the bulk plasma and energetic ions during an ALE and long after.

It is already possible to construct various hypothetical trigger scenarios for ALE-like relaxation events from combinations of the mechanisms discussed above: drive, damping and retardation effects, as well as changes in the topology of the magnetic field lines and particle trajectories. In order to test any such hypothesis, it would be extremely useful to simulate the spontaneous onset of an ALE during continuous MHD activity. We have recently reported progress in that direction^53^, but extensive case studies and reproducibility tests must still be completed before conclusive answers can be obtained. This work will be carried out using the petaflop-scale supercomputer JFRS-1 that is entirely dedicated to fusion science and began operation in June 2018 at QST Rokkasho Fusion Institute in northern Japan.

## Methods

### Code availability

Further information concerning the hybrid code MEGA^13,14,24^ and the version used in this work^25,28^ can be made available by the corresponding author upon reasonable request. The source code may be obtained after establishing an official research collaboration agreement with National Institutes of Quantum and Radiological Sciences (QST).

### Simulation model and parameters

A detailed description of the model equations, simulation setup and numerical techniques along with benchmarks, convergence tests and sensitivity studies can be found in previously published papers^17,25,28,29^. For the reader’s convenience, the information relevant here is summarised in the following paragraphs. A summary of relevant plasma parameters is given in Table 1.

**Table 1:** Plasma parameters in simulations based on JT-60U shot number E039672

Major radius	*R*_0_ = 3.4 m
Mean minor radius	〈*a*〉 ≈ 1 m
Plasma current	*I*_p_ = 0.57 MA
Toroidal field strength	*B*_0_ = 1.16 T (1.20 T)
Thermal/magnetic pressure ratio	*β*_0_ = 3.60% (3.37%)
Number density (ions)	*N*_i0_ = 1.74 × 10^19^ m^−3^
Number density (electrons)	*N*_e0_ = 2.23 × 10^19^ m^−3^
Temperature (ions)	*T*_i0_ = 1.28 keV
Temperature (electrons)	*T*_e0_ = 2.15 keV

The subscript 0 indicates that a quantity is measured at the centre (magnetic axis) of the plasma. The difference between the electron and ion densities is due to impurities (mainly carbon), which are not considered in our simulations. Two cases were simulated, which differ in the value of the toroidal magnetic field strength *B*_0_ and, thus, in the ratio of kinetic to magnetic pressure, which is measured by the plasma beta parameter *β*_0_. The values for the modified case are in parentheses

### Multi-phase simulation

In order to be able to simulate long intervals of tens and hundreds of milliseconds, as in Fig. 3, the MHD solver is turned on for only 1 ms during each 5 ms interval. A benchmark study was published in ref.^28^, where we have also pointed out that there must be (and, indeed, there is) some amount of overshoot in both the fluctuation amplitude and particle transport during the 1 ms periods with MHD. This compensates for the suppression of MHD during the preceding 4 ms interval.

### Monte-Carlo simulation

The dotted curve in Fig. 3g was also obtained using the MEGA code but with the MHD solver turned off at all times. Only beam injection and collisions were simulated. When run in this way, the version of MEGA used here is essentially identical to the OFMC code^22,23^, except for minor differences in the way the beam ions are deposited in the plasma^25^.

### Discretization

MEGA performs simulations using a finite-difference scheme in right-handed cylinder coordinates (*R*, *ϕ*, *Z*). The number of grid points used in the simulations reported here is *N*_*R*_ × *N*_*ϕ*_ × *N*_*Z*_ = 384 × 96 × 352, where we let *N*_*R*_ > *N*_*Z*_ because the magnetic surfaces are compressed in the region *R* > *R*_0_ on the outboard side of the plasma. The number of simulation particles accumulated by the end of the simulation is 7.4 million, which suffices^29^ for a beam ion distribution with a narrow range of pitch angles as seen in Fig. 4.

The equations are solved using the 4th-order Runge–Kutta scheme. The time step for the MHD solver is as small as 1 ns in order to satisfy the Courant-Friedrichs-Lewy condition for fast Alfvén waves. The time step for the PIC module is chosen 4 times larger than the MHD time step.

The values of the MHD coefficients controlling resistive, viscous and thermal diffusion are *η*/*μ*_0_ = *ν* = *χ* = 10^−6^*v*_A0_*R*_0_, which is considered to be a reasonable compromise between numerical and physical considerations^29^. At each time step, we filter out toroidal harmonics with *n* > 4 because—in the high-beta tokamak plasma considered here—these harmonics develop instabilities that bear features of resistive ballooning modes^17^. These modes can be stabilised by reducing the resistivity, but this would also require a higher spatial resolution with shorter time steps in order to keep numerical damping at a negligible level. Long-time simulations including *n* > 4 harmonics may, thus, require significantly larger computational resources (factor 8 or more).

### Data reduction

For post-processing, MEGA maps the raw data to toroidal flux coordinates (*ψ*, *ϑ*, −*ϕ*) and performs a Fourier decomposition into poloidal and toroidal harmonics with the basis function exp(*inϕ* + *imϑ*). This facilitates efficient data reduction as needed for recording snapshots at high sampling rates (here, 6–30 samples per Alfvén cycle). In the present simulations, we record harmonics with toroidal and poloidal mode numbers in the range −4 ≤ *n* ≤ 4 and 0 ≤ *m* ≤ 12. The results shown in Figs. 3a, b, 5 and 7a are obtained after recombining the 13 poloidal harmonics for each *n*. Meanwhile, Fig. 7b shows raw simulation data on the original mesh.

### Poincaré plots

The Poincaré plot in Fig. 7 were obtained by following the magnetic field lines using a 4th-order Runge–Kutta algorithm. The 91 starting points are located in the outer midplane at *ϑ* = *ϕ* = 0 and are equal spaced along the magnetic flux label *ψ* in the range 0 ≤ *ψ* ≤ 0.9. For each initial position, we recorded 450 Poincaré sections in the poloidal (*R*, *Z*) plane at *ϕ* = 0. For clarity, Fig. 7e, f shows Poincaré sections only for every third starting point along *ψ*. Fig. 7c shows the complete data.

### Experimental measurements

Information concerning the experimental setup and the conditions under which Abrupt Large-amplitude Events (ALE) were observed can be found in refs.^4,6,7,25^. As noted in ref.^25^, JT-60U shot number E048424 examined in Fig. 6 belongs to a more recent campaign that followed an upgrade of a digitizer for the magnetic sensors (E044000 and after). For instance, the upgrade allowed to increase the sampling frequency for the saddle loop coil system from 40 kHz to 500 kHz, so that it became possible to distinguish different toroidal harmonics with *n* = 0–3 in the ALE-relevant frequency range 30–70 kHz.

Shot E048424 was originally performed to study other phenomena, and here we have analysed the ALEs in that experiment for the first time. For Fig. 6, we have chosen saddle loop signals because they offer a better signal-to-noise ratio, owing to the large surface area of each coil. This comes at the expense of lower sensitivity for shorter wavelengths (that is, larger mode numbers *n* and *m*), and we believe that this reduced sensitivity is the main reason for why the *n* = 3 signal in Fig. 6 is significantly weaker than the *n* = 1 and *n* = 2 signals. Two of the 8 saddle loops cover as much as 40° in the toroidal direction; that is, 33% of the *n* = 3 wavelength. The poloidal extent of all coils is 1.35–1.37 m, so they average over about 10–15% of the plasma circumference in the poloidal direction. This means that most of the poloidal harmonics *m*≈*nq* in the range 4–12 for *n* = 3 and *q*≈1.3–4 are strongly attenuated or invisible. The data in Fig. 6 were not corrected for their wavelength-dependent sensitivity, because this would require making some assumptions about the mode structure.

We have made attempts to use Mirnov coil signals in order to improve the quality of the *n* = 3 signal in Fig. 6 and obtain information for *n* = 4, but without success. The Mirnov coil array gave too few and too nonuniformly spaced data points. There were 9 Mirnov coils, but only 6 of them produced meaningful signals. *n* = 1 and *n* = 2 signals are robust, but the *n* = 3 signal is uncertain because the 6 Mirnov coils that produced useful data are very nonuniformly spaced. Nonuniform spacing can be used to measure short wavelength with few coils, but only if signals of different wavelengths also have sufficiently different frequencies. In the case of ALEs, the mode frequencies are too similar, so that nonuniformly spaced coils cannot be used to determine how much energy is contained in different wavelengths, especially for *n* > 2. In the present case, this kind of aliasing causes the amplitude of the *n* = 3 harmonics to vary by factors of 3–4 depending on how the nonuniform data was interpolated or weighted.

### Data availability

The data that support the findings of this study are available on request from the corresponding authors (A.B. for simulations, K.S. for experiments). The data are not publicly available due to regulations of National Institutes for Quantum and Radiological Science and Technology (QST). Before the data can be released, an official research collaboration agreement with QST must be established.

## Electronic supplementary material

Peer Review
