# Supplementary material for: Simulations tackle abrupt massive migrations of energetic beam ions in a tokamak plasma
Source: Nat Commun. 2018 Aug 16;9:3282. doi: 10.1038/s41467-018-05779-0 (PMC6095890; doi:10.1038/s41467-018-05779-0)
Supplement: Supplementary file 1 — Peer Review [file 41467_2018_5779_MOESM1_ESM.pdf]

Reviewers' comments:

Reviewer #1 (Remarks to the Author):

This paper presents progress on simulating Abrupt Large-amplitude Events (ALE) which are sudden, fast timescale events that undesirably redistribute high energy (500 keV) fast ions in fusion plasmas. The new work is that the MEGA code has been used to simulate multiple ALE cycles, and the simulations have been run to allow the computation of multiple toroidal mode numbers which shows that ALEs have multiple toroidal mode numbers that nonlinearly interact. Measured magnetic signals are also found to be composed of multiple toroidal mode numbers, supporting the simulation findings. There are a number of items that need to be clarified throughout the text before the paper can be published. The paper would be more influential if the authors could extract more information from the simulation on the physics of when and why ALEs occur.

1. The abstract says “In order to unravel the underlying physical processes and predict whether similar intermittent migrations of energetic ions will occur in future fusion experiments and reactors, it is necessary to simulate these complex multi-time-scale dynamics numerically. Here, we present results from simulations that have successfully accomplished this task and reproduced multiple migration cycles.” On first read, the clause “successfully accomplished this task” can be interpreted as successfully unraveling the underlying physics and doing predictions as described in the previous sentence, which has not been done here. In order to avoid over-selling, the last sentence should be changed to “Here, we present results from simulations that have successfully reproduced multiple migration cycles.”

2. In Figure 2, the schematic representation of the plasma torus is odd. What do the red dots in the plasma represent? Is the yellow the background thermal plasma and the red dots the beam ions? Is it turbulent electric field fluctuations? On the right hand figure, it would be more meaningful to show the toroidal mode structure of magnetic field fluctuations, since that is what is being compared to measurements.

3. (Page 3, line 4). It would be helpful to give more explanation of the term “transit resonances”

4. (Page 3, line 10). It is not clear what all is meant by “pre-defined initial conditions”. Do you mean that experimental plasma profiles and magnetic equilibrium are used as initial conditions?

5. (Page 4, line 2) By “arbitrary initial condition”, do you mean arbitrary energetic ion distribution? Aren’t experimental plasma profiles needed to initiate the simulation?

6. (Page 5, line 3) The physics motivation for simulating two different magnetic fields should be stated upfront here. Why is BT only changed by ~3%. Why not change the field more than that?

7. (Page 5, line 19) It is not clear what is meant by “overshoot” in the sentence “Except for the MHD activity being turned off artificially at 5 ms intervals, which can result in some amount of overshoot,…” Overshoot of what?

8. (Page 6, line 8). You seem to argue that theoretically, the ALE is expected to appear when a specific value of the spatial gradient in beta_E is reached. In Figure 3b, why is the peak value of beta_E at the time of the first ALE lower than it is for the second or third ALE? Is there a difference in the energetic particle distribution and plasma parameters at the time of the first ALE compared to the second and third ALEs? Is the peak value of the spatial gradient in beta_E the same at the time that each ALE occurs?

9. (Page 6, line 18) It is not clear what is meant by the last two sentences. Can you expand on what you mean by doing a quantitative test? By “expect the observed trend is enhanced”, do you mean the difference in timing of the ALEs between the two B field comparisons would change?

10. Figure 3c. The magnetic axis should be marked to more readily identify the core.

11. (Page 7, line 1) Technically you are showing energetic ion beta, not pressure.

12. (Page 7, line 4) When you say “a conventional Monte-Carlo particle simulation” are you still using the same MEGA code except with MHD activity turned off? The way it is written makes it sound like it is a simulation from a different code.

13. (Page 7, Section 4). Can the authors expand on how the results of this simulation be used to construct reduced models?

14. (Page 7, Section 4). How many poloidal harmonics were included?

15. (Page 8 line 6 and line 7) where→were

16. (Page 10, line 16) What is meant by “Such simulations allow us to compute the distribution of energetic ions with one order of magnitude better accuracy…” How do you quantify the accuracy of the distribution function, since the full distribution function is not measured experimentally?

Reviewer #2 (Remarks to the Author):

The authors have performed the hybrid gyrokinetic-MHD simultion on ~100 ms time scale to reproduce the whole cycle of the Abruptive Large-amplitude Events (ALE) observed at the Japanese tokamak JT-60U. The authors also demonstrate the important underlying physics during this intermittent nonlinear process. The topic is cruicial for improving the fusion product confinement in tokamak devices. The robustness of the numerical method, the realistic physics model for the neutral beam source and collisions, and the dedicated supercomputer resources are crucial for the complishment of this work. Considering the solid evidence for the conclusion, the novelty in terms of the simulation time scale, and the potential impact on transport modelling and intermittent nonlinear phenomenon understanding, I recommend the acceptance of this manuscript after minor revision according the following comments.

1. In Page 4, the authors mentioned that ``if one is interested only in an estimate of the steady-state pressure field of the energetic ions when beam deposition and transport are more or less in balance, it can be sufficient to simulate MHD activity for only 1 ms out of every 5-–10 ms period'. How valid is this condition during the whole stage of the ALE simulation. In particular, when ALE occurs, beam transport is much larger than the rest of time, as shown in Fig. 3 (b), then should MHD solver always be turned on during this period? If not, what has been done to make sure that the realistic physics is kept? Since the simulated ALE last for around $1s$ as shown in Fig. 4, I suggest to mark when the MHD solver is switched on during ALE.

2. While it is not practical to give all the details of the simulation, necessary information can be mentioned, e.g., the shot number or references where detailed parameters are reported. In particular, how far away is the beam gradient from the critical gradient? What are the features of safety factor,

density, temperature and rotation profiles?

3. In Page 6, while the energetic ion beta rises earlier for the low-field case (1.16T), the following time evolution shows the opposite trends. Namely, from the first maximum value of beta to the second one, and from the second peak to the third one, it takes shorter for the low-field case. How does the nonlinear process change the evolution of the the beam ion beta after the first peak?

4. In Page 7, there is some ambiguity when the author mentioned the ``long-standing issue" in Reference [29] and then stated that simulations reported in their work offer a way to resolve this problem, at the expense of consuming nearly 100 times larger computational resources. How are the simulations in this work relevant to the fast particle flattening observed in experiment of Reference [29], considering the coexistence of toroidicity-induced, reversed-shear Alfven eigenmodes and many weaker intermittent modes appear at lower frequencies in [29]? Does the author indicate that the weaker intermittent modes appear at lower frequencies in [29] can be reproduced by their simulations? Another confusion is the ratio number ``100 times' since the NOVA code and ORBIT code mentioned in [29] can consume much less than 1% of the computation time in this work. I would assume that authors are trying to compare the computational resource consumption of short time scale simulation using hybrid code, e.g., in [15], with the simulations in this work. Please clarify it.

5. In Fig. (4c), it is suggested that the synergy of n=2 and n=3 modes can be important for triggering the ALE. However, as the authors noticed, and mentioned in Page 11, more work is needed for the the fully understanding of the mechanism. While the present results already demonstrate the important features of ALE, I suggest performing analysis using the available data to clarify several issues. For example, the Lissajous curves can help identify the nonlinear coupling between different modes (e.g., [T. Ido, Phys. Rev. Lett. 116, 015002 (2016)]), namely, the n=1,2,3 modes in this work, for simulation data or experiment data. Also, if the particle data is available, it is instructive to mention the particle resonance region (e.g., [Todo, Nucl. Fusion 54 (2014) 104012, H. S. Zhang, Phys. Rev. Lett. 109, 025001 (2012); X. Wang, Phys. Rev. E. 86, 045401(R) (2012)]) when different combination of n modes is kept and it will be a compensation to Fig. 4. It will also be useful information for stimulating theoretical and modeling work by others.

Reviewer #3 (Remarks to the Author):

The manuscript presents results of multiscale numerical simulations aimed to reproduce so-called “Abrupt Large-amplitude Events (ALE)” in JT-60U tokamak.The present study differs from previous studies by the same authors, in that the simulation model now includes the ion sources and collisions, allowing a self-consistent simulation of the accumulation and distribution of energetic ions in the presence of MHD perturbations. Very time consuming and expensive simulations are performed, therefore, without making any assumptions regarding the energetic ion distribution. I believe that the manuscript is mostly suitable for publication, but I do have several minor comments and suggestions for improvement:

1. The authors describe large-amplitude events, but there is no information in the manuscript regarding the observed amplitudes.

2. In section 3, the author’s description of how exactly “the MHD ‘solver’ was turned on at 5ms intervals” is not clear, and a better description is needed.

3. p.7 should mention the experimental results regarding multi-mode nature of the observed ALEs.

4. On p.8 the authors say that the mode with n>4 were not well resolved in simulations. What was the grid resolution (toroidal) in simulations, number of simulation particles? Have the authors performed any convergence tests?

5. In Fig.4 – modes with n=1-3 show comparable growth times and amplitudes, could it be related to numerical coupling between modes with different n? Also, there is no distinct linear growth phase in Figs. 4b and 4c – how the numerical noise level compares with mode saturation amplitudes?

6. Fig.3 and its description – how the mode amplitudes in simulations compare with the experimental results?

7. Fig. 5 – description says that the experimental data was decomposed into n=1-3 Fourier harmonics. Is there any evidence that the higher n's were not present in the experiments?

8. It would helpful for the reader, if the descriptions of the results given in the Figure Captions were moved (or duplicated) in the text of the manuscript.

Title: Simulations tackle abrupt massive migrations of energetic beam ions in a tokamak plasma

Authors: A. Bierwage, K. Shinohara, Y. Todo, N. Aiba, M. Ishikawa, G. Matsunaga, M. Takechi and M. Yagi

We thank the three referees for carefully checking and evaluating our manuscript. The referees' detailed reports and constructive comments were highly appreciated as they helped us to improve the paper significantly in terms of clarity, accuracy and completeness. Below are our answers to the questions, comments and suggestions we received, which are *cited in blue italics*. The answer letter is followed by a complete list of changes, and a copy of the manuscript where all important changes are highlighted.

Answers to comments by Referee 1

0. *[...] The paper would be more influential if the authors could extract more information from the simulation on the physics of when and why ALEs occur.*

   We agree and since the word count limit ($\sim$ 5000) for Nature Communications articles is larger than the original submission, there was room left for expansion that we have filled with additional results (Figs. 4 and 7) and corresponding discussions. The new additions are indicated in blue colour in the annotated version of the revised manuscript and may be outlined as follows:

   - Discussion of the difference between the first and subsequent ALEs (page 7, lines 16ff).
   - Discussion of the restoration of the pressure profile and velocity distribution, and the role of collisional slow-down (page 10, line 10 until page 11; new Fig. 4).
   - Observation of nonlinearly driven magnetic reconnection (page 14, line 10 until page 16; new Fig. 7).

   We hope that this enhances the impact of our paper.

1. *The abstract says "In order to unravel the underlying physical processes and predict whether similar intermittent migrations of energetic ions will occur in future fusion experiments and reactors, it is necessary to simulate these complex multi-time-scale dynamics numerically. Here, we present results from simulations that have successfully accomplished this task and reproduced multiple migration cycles." On first read, the clause "successfully accomplished this task" can be interpreted as successfully unravelling the underlying physics and doing predictions as described in the previous sentence, which has not been done here. In order to avoid over-selling, the last sentence should be changed to "Here, we present results from simulations that have successfully reproduced multiple migration cycles.".*

   We agree and have modified the abstract to eliminate the ambiguity.

2. *In Figure 2, the schematic representation of the plasma torus is odd. What do the red dots in the plasma represent? Is the yellow the background thermal plasma and the red dots the beam ions? Is it turbulent electric field fluctuations? On the right hand figure, it would be more meaningful to show the toroidal mode structure of magnetic field fluctuations, since that is what is being compared to measurements.*

   Indeed, the yellow was meant the represent the background thermal plasma, and the red dots the beam ions. However, we realized that the figure was misleading, e.g., because it suggested that the density of energetic particles is similar to that of bulk particles, which is not true. We have replaced this figure.

   Concerning the mode structure, which now appears at the top right of the revised figure 2, we think that E_ϕ is actually a useful representation of the Alfvénic fluctuations because magnetic and electric perturbations are closely linked for such (nearly) ideal MHD waves. The fluctuating magnetic vector field $\delta\boldsymbol{B}$ is difficult to visualize directly in an intelligible way. However, in a tokamak plasma we can make use of the fact that the magnetic perturbations have the form $\delta\boldsymbol{B} \approx \nabla\delta\Psi \times \hat{\boldsymbol{e}}_\phi$ and plot contours of the scalar flux function $\delta\Psi$ instead of the vector field. Assuming the time dependence $e^{-i\omega t}$, the Maxwell-Faraday equation $\partial_t\boldsymbol{B} = -\nabla\times\boldsymbol{E}$ implies that $i\omega\delta\Psi \approx \delta E_\phi$. Thus, except for factors ω and n, we think

that contour plots of $\delta E_\phi = -\partial_\phi \delta\Phi = \sum_n in\delta\Phi_n e^{-in\phi}$ offer a compact and intelligible representation of the perturbed flux function $\delta\Psi$ or electrostatic potential $\delta\Phi$, which are closely related in ideal MHD. (Please excuse the simplified notation.)

3. *(Page 3, line 4). It would be helpful to give more explanation of the term "transit resonances".*

   We have added an explanation on page 3, lines 3–9.

4. *(Page 3, line 10). It is not clear what all is meant by "pre-defined initial conditions". Do you mean that experimental plasma profiles and magnetic equilibrium are used as initial conditions?*

   Pre-defined initial conditions based on JT-60U plasma parameters are used in previous and present simulations. In the new simulations, the arbitrary initial conditions for the energetic ion distribution were eliminated. We realize that the sentence pointed out by the referee is confusing since the distinction between similarities and differences becomes unclear, so we removed the statement about pre-defined initial conditions from this paragraph (now line 16 on page 3).

5. *(Page 4, line 2) By "arbitrary initial condition", do you mean arbitrary energetic ion distribution? Aren't experimental plasma profiles needed to initiate the simulation?*

   Yes, this phrase was meant to refer to the energetic ion distribution, which was previously modeled using mathematical formulas or, at best, using results from "classical" orbit-following Monte-Carlo (OFMC) simulations. These models contained free parameters, which could be set arbitrarily. Even when using computed "classical" distribution functions, a free scaling factor was usually used to correct the overestimated beta value. We have clarified this point by rephrasing this part as: "In order to eliminate the need to predefine or tune the energetic ion distribution and make progress toward actual predictions [...]" (page 4, line 16). Before this sentence (also on page 4), we have also added a paragraph describing what kind of tuning had been used before (lines 3–9).

6. *6. (Page 5, line 3) The physics motivation for simulating two different magnetic fields should be stated upfront here. Why is $B_{\rm T}$ only changed by $\sim 3\%$. Why not change the field more than that?*

   The reason is mainly a historical one: The stability and short-time response of these two cases were studied intensively in previous works, so we are already fairly familiar with their properties. We understand that this is a somewhat conservative choice. Broader parameter scans would be desirable. However, computational resources are limited. As an afterthought, taking a positive stance, we think that the comparison between these two similar cases can be regarded as a sensitivity test because parameters were changed, but at the same time it is also a reproducibility test because parameters were not changed dramatically (page 7, lines 5–8).

   While revising the manuscript, we have noticed that we had forgotten to mention that not only the toroidal magnetic field strength B_0 was changed but also the birth energy $W_0 = m_{\rm D}v_0^2/2$ of the energetic ions. In fact, the following two dimensionless parameter has the same value in both simulations:

   - beam-to-Alfvén velocity ratio: $v_0/v_{\rm A0} = 1.432$.

   In order to make these points clear, we have expanded the paragraphs where the parameter settings are explained: from page 5, line 13 until page 6, line 9.

7. *(Page 5, line 19) It is not clear what is meant by "overshoot" in the sentence "Except for the MHD activity being turned off artificially at 5 ms intervals, which can result in some amount of overshoot,..." Overshoot of what?*

   There is (and there must be) an overshoot in both mode amplitude and transport in order to compensate for the lack of both during the intervals where MHD activity was suppressed. We agree that this was not clearly communicated in our short sentence. Since this point was already explained in [Bierwage *et al.* *Comp. Phys. Comm.* **220** (2017) 279] and since it is not essential here, we decided to remove it from the main text to reduce distraction and avoid confusion. The paragraph was moved to the new "Methods" section on page 20, lines 2–7.

8. *(Page 6, line 8). You seem to argue that theoretically, the ALE is expected to appear when a specific value of the spatial gradient in $\beta_{\rm E}$ is reached. In Figure 3b, why is the peak value of $\beta_{\rm E}$ at the time of the first ALE lower than it is for the second or third ALE? Is there a difference in the energetic particle distribution and plasma parameters at the time of the first ALE compared to the second and third ALEs? Is the peak value of the spatial gradient in $\beta_{\rm E}$ the same at the time that each ALE occurs?*

   Actually, we do not think that a scalar quantity like $\beta_{\rm E}$ and not even its radial profile will suffice as an accurate indicator for critical gradients, because we expect that this would work well only in two limits: (a) there is only one mode and only one resonance, or (b) there is a broad spectrum of modes and many resonances. The present intermediate case with a few modes is probably less predictable with a low-parametric model, and one would have to look at the situation near the dominant resonances in phase space, as is indeed done in advanced critical gradient models [e.g., Collins *et al. Nucl. Fusion* **57** (2017) 043001]. We mention this point on page 10 (lines 3–9) of the revised manuscript.

   Nevertheless, we thought to see at least a rough $\beta_{\rm E}$-dependent trend in the comparison between the two cases. But it seems that we did not look carefully (or not objectively) enough at the data and agree that our discussion of this point was flawed in several ways, as all referees have correctly noticed. Therefore, we have removed this discussion from the revised manuscript.

   As a substitute, and motivated by one of the referee's questions, we added a new discussion concerning the difference between the first and all subsequent ALEs on page 7 (lines 16ff) of the revised manuscript. The first ALE is indeed different from all subsequent ones:

   (i) When the first ALE occurs, the fast ion tail has just barely filled the resonant phase space. All later ALEs occur in the presence of a fully developed energetic ion tail. For instance, see Fig. 4 in [Bierwage *et al. Nucl. Fusion* **54** (2014) 104001] or Fig. 1 in [Bierwage *et al. Comp. Phys. Comm.* **220** (2017) 279]. Actually, due to the influence of preceding ALEs, it becomes a bump-on-tail distribution as shown in the new Fig. 4.

   (ii) Each ALE dumps particles into the plasma periphery, so the energetic ion beta profile $\beta_{\rm E}$ expands radially before narrowing down again since the particles in the cooler periphery thermalize more rapidly than those in the hotter core. Before the first ALE, the $\beta_{\rm E}$ profile does not go through this narrowing-down process.

   Due to these factors, we believe that the timing of the first ALE is delayed and possibly more sensitive to the history of low-amplitude fluctuations than later ALEs. A discussion of this point has been added in the revised manuscript. Please see the new paragraph on page 7 (lines 16ff) and the new subsection titled "Restoration of pressure profile and velocity distribution", which can be found on pages 10–11, along with a newly added Fig. 4.

9. *(Page 6, line 18) It is not clear what is meant by the last two sentences. Can you expand on what you mean by doing a quantitative test? By "expect the observed trend is enhanced", do you mean the difference in timing of the ALEs between the two B field comparisons would change?*

   This part was removed due to the issues mentioned in item 8 above.

10. *Figure 3c. The magnetic axis should be marked to more readily identify the core.*

    Done.

11. *(Page 7, line 1) Technically you are showing energetic ion beta, not pressure.*

    This is correct, but we chose the word "pressure" to communicate more clearly the meaning of the results for readers from different disciplines. This sentence discusses the temporal evolution of the beta profile. Since the magnetic field does not change (in the sense that $\delta B \ll B_0$) the variation in beta represents a variation in pressure only.

12. *(Page 7, line 4) When you say "a conventional Monte-Carlo particle simulation" are you still using the same MEGA code except with MHD activity turned off? The way it is written makes it sound like it is a simulation from a different code.*

    The collision and beam deposition model used in the present version of MEGA was adopted from the OFMC code as described in [Bierwage *et al. Nucl. Fusion* **57** (2017) 016036]. Thus, although we obtained these results using "MEGA without MHD" this would be essentially the same as saying that the classical profile was computed using the OFMC code. We believe

that some ambiguity is tolerable here, so we left that sentence as is for the sake of simplicity. Nevertheless, we did add a paragraph in the new "Methods" section on page 20 (lines 8-11), where we discuss the relation between MEGA and OFMC.

13. *(Page 7, Section 4). Can the authors expand on how the results of this simulation be used to construct reduced models?*

    We are not prepared to make concrete suggestions and can only point out the possibility and a few factors that should be taken into consideration. In the revised manuscript, we have included such discussions in the form of interludes that connect some of the subsections under "Results":

    - Last paragraph of the subsection on "Massive migration [...]": Page 10, lines 3–9.
    - Last paragraph of the new subsection on "Restoration [...]": Page 11, lines 15–18.

    In addition, in the "Discussion" section on page 17 (lines 13–16), we state: "Further work is required in order to fully unravel the mechanisms that trigger ALE-like relaxation events and to become able to construct computationally efficient yet reliable reduced models. The analysis of the simulated ALE dynamics is underway and first new insights were reported here."

14. *(Page 7, Section 4). How many poloidal harmonics were included?*

    MEGA performs simulations using a finite-difference scheme in cylinder coordinates (R, ϕ, Z). Decomposition into poloidal harmonics is performed only as a post-processing step for data reduction, which is needed to write out snapshots with a high sampling rate. The procedure is now described in the new "Methods" section on pages 20 (lines 12–17) and 21 (lines 9–15) of the revised manuscript.

15. *(Page 8 line 6 and line 7) where → were*

    Corrected. Thank you.

16. *(Page 10, line 16) What is meant by "Such simulations allow us to compute the distribution of energetic ions with one order of magnitude better accuracy..." How do you quantify the accuracy of the distribution function, since the full distribution function is not measured experimentally?*

    Indeed, this statement cannot be supported by direct comparison in the present case. One factor that led us to estimate the improvement as "one order of magnitude better accuracy" was the recent validation of MEGA simulation results against measurements made in the DIII-D tokamak, where improvements at that level were achieved. In order to clarify this point, we have expanded the discussion of the prediction accuracy, which now begins on page 16, line 17.

Answers to comments by Referee 2

1. *In Page 4, the authors mentioned that "if one is interested only in an estimate of the steady-state pressure field of the energetic ions when beam deposition and transport are more or less in balance, it can be sufficient to simulate MHD activity for only 1 ms out of every 5-10 ms period". How valid is this condition during the whole stage of the ALE simulation. In particular, when ALE occurs, beam transport is much larger than the rest of time, as shown in Fig. 3(b), then should MHD solver always be turned on during this period? If not, what has been done to make sure that the realistic physics is kept? Since the simulated ALE last for around 1s [→ 1 ms] as shown in Fig. 4, I suggest to mark when the MHD solver is switched on during ALE.*

   To begin with a short answer: The MHD solver is switched on for the entire time interval shown in Fig. 5 (former Fig. 4). This can be inferred from the fact that the fields fluctuate in these time traces. We have added an explicit statement in the caption of Fig. 5 (line 4).

   Here are some more details: The radial mixing of energetic particles is essentially finished when the peak of the ALE is reached. After that the waves decay and the 3-dimensionally (in space) perturbed particle distribution equilibrates into a quasi-1D ("radial") profile. Figures 5 (sim.) and 6 (exp.) in the revised manuscript (formerly Figs. 4 and 5) as well as previous experimental studies show that the ALE lasts less than 1 ms, so we may assume that a 1 ms

long hybrid simulation (with MHD) is sufficient to capture one event.

After we had identified ALE events in the "1 ms MHD on + 4 ms MHD off" simulations shown in Fig. 3, we have chosen one such event (here $t = 129\,\mathrm{ms}$) and repeated the simulation of this ALE with various combinations of toroidal harmonics. The results of these test runs are shown in Fig. 5 (formerly Fig. 4). The MHD solver was switched on for the entire duration of these restarted simulations, which ran for up to 2 ms. Figure 4 shows the MHD activity during the first 1.5 ms of these simulations. This is to show that ALE-like events do not occur during that time interval when only a single n harmonic is included (and also for certain pairs of n numbers).

2. *While it is not practical to give all the details of the simulation, necessary information can be mentioned, e.g., the shot number or references where detailed parameters are reported. In particular, how far away is the beam gradient from the critical gradient? What are the features of safety factor, density, temperature and rotation profiles?*

   We have expanded the first second paragraph of the "Results" section to include information about the shot number and simulation parameters. Please see page 5, line 19 until page 6, line 15 of the revised manuscript. Further information about the plasma parameters is given in Table 1 of the "Methods" section (page 31). Although it appears rather late in the revised manuscript, the safety factor profile $q(\psi)$ is now shown in panel (d) of the newly added Fig. 7. We call it "magnetic field line pitch (= tokamak safety factor)" in order to make its meaning clear for readers from other disciplines. Rotation is not mentioned because it is only a few kHz; i.e., negligible compared to the high-frequency Alfvén modes in the 30–70 kHz band that are relevant for ALEs.

   Concerning the "critical gradient", we do not think that such a quantity can be defined for the present system in a straightforward manner. In the previous version of the manuscript, our discussion concerning the role of β_{E} was probably misleading in that regard. There were some flaws in that discussion, so we have removed it. Please see also our reply to the closely related comment 8 by Referee 1. An advanced critical gradient model is also mentioned on page 10, lines 4–5.

3. *In Page 6, while the energetic ion beta rises earlier for the low-field case ($1.16\,\mathrm{T}$), the following time evolution shows the opposite trends. Namely, from the first maximum value of beta to the second one, and from the second peak to the third one, it takes shorter for the low-field case. How does the nonlinear process change the evolution of the beam ion beta after the first peak?*

   We appreciate the referee's pointing out these issues and realize that our discussion of the results was flawed. This discussion was removed and replaced with a new discussion concerning the difference between the first and all subsequent ALEs on page 7 (lines 16ff) of the revised manuscript. Please see also our reply to the closely related comment 8 by Referee 1.

4. *In Page 7, there is some ambiguity when the author mentioned the "long-standing issue" in Reference [29] and then stated that simulations reported in their work offer a way to resolve this problem, at the expense of consuming nearly 100 times larger computational resources. How are the simulations in this work relevant to the fast particle flattening observed in experiment of Reference [29], considering the coexistence of toroidicity-induced, reversed-shear Alfven eigenmodes and many weaker intermittent modes appear at lower frequencies in [29]? Does the author indicate that the weaker intermittent modes appear at lower frequencies in [29] can be reproduced by their simulations? Another confusion is the ratio number "100 times' since the NOVA code and ORBIT code mentioned in [29] can consume much less than 1% of the computation time in this work. I would assume that authors are trying to compare the computational resource consumption of short time scale simulation using hybrid code, e.g., in [15], with the simulations in this work. Please clarify it.*

   This part of the text was indeed not written clearly. We were trying to say that a "classical" OFMC simulation of the same case would be 100 faster than the current MEGA simulation, but the results are off by a factor 2 or so. The fact that "classical" predictions can have large discrepancies is long known, and one place were this was discussed is Ref. [29] (although the analyses in Ref. [29] go well beyond the classical limit).

   In order to improve clarity, we have expanded that paragraph, which now appears on page 9, lines 7–18. There we also mention that codes using reduced (low-dimensional or *ad hoc*) transport models can run much faster than OFMC.

5. *In Fig. (4c), it is suggested that the synergy of $n = 2$ and $n = 3$ modes can be important for triggering the ALE. However, as the authors noticed, and mentioned in Page 11, more work is needed for the the fully understanding of the mechanism. While the present results already demonstrate the important features of ALE, I suggest performing analysis using the available data to clarify several issues. For example, the Lissajous curves can help identify the nonlinear coupling between different modes (e.g., [T. Ido, Phys. Rev. Lett. 116, 015002 (2016)]), namely, the $n = 1, 2, 3$ modes in this work, for simulation data or experiment data. Also, if the particle data is available, it is instructive to mention the particle resonance region (e.g., [Todo, Nucl. Fusion 54 (2014) 104012, H.S. Zhang, Phys. Rev. Lett. 109, 025001 (2012); X. Wang, Phys. Rev. E. 86, 045401(R) (2012)]) when different combination of n modes is kept and it will be a compensation to Fig. 4. It will also be useful information for stimulating theoretical and modeling work by others.*

   Motivated by discussions with the author of [M. Lesur, Phys. Rev. Lett. 116, 015003 (2016)], Lissajous analyses for these simulations have been carried out a few years ago and were reported at ITPA Technical Meeting on Energetic Particles in 2016. However, the results were inconclusive. No clear correlations could be identified between different modes. Indeed, this negative result may be expected from the following consideration:

   - Lissajous figures allow to detect correlations between two modes with low-order rational frequency ratios. The correlation should preferably last for many cycles in order to be clearly distinguishable. However, in our case, three modes grow to large amplitudes almost simultaneously, so pairwise correlations may be easily disturbed by the third player. Moreover, the frequencies for $n = 1, 2, 3$ are around 50, 45 and 55 kHz, respectively, which means that a Lissajous figure would form only after 4 oscillation cycles or more, and multiples of that would be needed to distinguish correlated and uncorrelated times. Thus, even if there were (direct or indirect) mode-mode couplings, they could not be spotted in Lissajous figures because the different stages of an ALE (onset, growth, saturation, decay) last only a couple of wave oscillation cycles each.

   We have revised and expanded our discussion of mode coupling and resonance overlap effects on pages 17–19 of the revised manuscript, and conclude with the statement saying that "Untangling these interactions during a short event like an ALE is a difficult task and a subject of ongoing research" (page 19, lines 2–3).

Answers to comments by Referee 3

1. *The authors describe large-amplitude events, but there is no information in the manuscript regarding the observed amplitudes.*

   On page 2 (lines 13–14), we have added a sentence saying that the ALE amplitudes are typically 10 times larger than the amplitudes of fluctuations at other times. On page 7 (lines 9–15), we have added a paragraph where we mention the absolute amplitudes in experiment and simulations, while emphasizing that a direct comparison is not possible since experimental measurements are external (near the wall) and numerical ones are internal (well inside the artificial fixed boundary).

2. *In section 3, the author's description of how exactly "the MHD 'solver' was turned on at 5ms intervals" is not clear, and a better description is needed.*

   We agree that some important information about the method was missing. On page 4, lines 18ff, we have added: "During the intervals where the MHD solver is turned off, all field fluctuations are set to zero, so the energetic ion population evolves only under the influence of collisions, sources and sinks in the equilibrium magnetic field. Each time the MHD solver is turned on, the fluctuations start from zero." Further information is given in the new "Methods" section under "Multi-phase simulation" on page 20 (lines 2–7).

3. *p.7 should mention the experimental results regarding multi-mode nature of the observed ALEs.*

   Until recently, it was widely believed that ALEs were due to so-called energetic particle modes (EPM) with $n = 1$. The low-amplitude fluctuations between ALEs were thought to be due to Alfvén eigenmodes. There was no experimental evidence to suggest otherwise. The multi-mode nature was first predicted in recent numerical simulations and resonance analyses (see

page 12, lines 8–13). Motivated by those predictions as well as the new long-time simulation results reported here, we searched the JT-60U database for evidence as stated a little later, now on page 13 (lines 11ff) of the revised manuscript. This experimental evidence is reported in this paper for the first time.

4. *On p.8 the authors say that the mode with $n > 4$ were not well resolved in simulations. What was the grid resolution (toroidal) in simulations, number of simulation particles? Have the authors performed any convergence tests?*

   At the beginning of the section titled "Simulations reproduce sequences of abrupt large events" (now on page 5, lines 13–18), we have cited several references where we "justified the simulation parameters, benchmarked our code and numerical scheme, and validated the simulation results against other experimental observations." For the reader's convenience, we have added information about the spatial resolution and the number of particles in the new "Methods" section under "Discretization" on page 20 (lines 12ff).

   Although we did not write that "$n > 4$ were not well resolved", we see how our wording "may not be adequately resolved" can easily be misunderstood. What we meant to say is that we did not verify whether the resolution suffices also for $n > 4$. In order to avoid misunderstandings in the revised manuscript, we simply write that "harmonics with $n = 5$ and larger were filtered out because they do not seem to be crucial here" (page 12, lines 12–13).

   Because we consider $n > 4$ harmonics to be not essential, we have used only $N_\phi = 96$ grid points in the toroidal direction. This ensures that $n = 4$ is well resolved. At first glance, 96 grid points may look like enough for toroidal harmonics as high as $n = 96/8 = 12$. However, this is not certain in the nonlinear regime, as we found in convergence tests performed as early as 2013, which are reported in [Bierwage *et al.* *Nucl. Fusion* **54** (2014) 104001] as originally cited at the end of the sentence pointed out by the referee. Please see p. 3 of that reference [17]. There it was shown that the linear growth of the $n < 5$ modes can be captured accurately with 32 toroidal grid points (even 16 points would be fine). However, we also found that higher resolution is required for an accurate simulation of nonlinear dynamics, such as the threshold for the onset of so-called convective amplification (CA), which was one of the main topics of that paper and may also play a role for triggering the ALEs studied here. The threshold for convective amplification of the $n = 3$ mode seemed to be reasonably converged with $N_\phi = 48$, and we increased this value to 96 in order to be on the safe side with $n = 4$.

   One reason for why we filter out higher-n harmonics is that they become increasingly sensitive to resistive and viscous dissipation. As we have shown in [Bierwage *et al.* *Nucl. Fusion* **54** (2014) 104001], in the present high-beta tokamak plasma the higher-n harmonics develop instabilities that bear features of resistive ballooning modes. These would disappear if we reduce the resistivity, but this would also require us to increase the spatial resolution and reduce the time step in order to keep numerical damping at a negligible level. The present long-time simulations would then become infeasible. This point is now also mentioned under "Discretization" in the new "Methods" section (see page 21, lines 1–8).

5. *In Fig. 4 — modes with $n = 1 - 3$ show comparable growth times and amplitudes, could it be related to numerical coupling between modes with different n? Also, there is no distinct linear growth phase in Figs. 4b and 4c — how the numerical noise level compares with mode saturation amplitudes?*

   In Fig. 5 (former Fig. 4) the noise level is clearly indicated as a shaded region labeled "PIC noise". The ALE peak amplitude is a factor 50 above the noise. Without ALE, the saturation levels are a factor 5 above noise. Of course, the modes will decay back to noise level whenever the resonant drive is exhausted and resonant phase space structures are destroyed under the influence of dissipation, collisions and to some extent also PIC noise.

   It is difficult to obtain a distinct linear growth phase with a 5-D full-f PIC simulation as performed here, unless one uses an extremely large number of particles. For the purpose of the present work, we think that it is acceptable to do without a distinct linear phase.

   If one looks closely at the few 100 microseconds before the ALE, one can see that the modes evolve rather independently initially. Perhaps this is not easy to see in the logarithmic plots in Fig. 5 (former Fig. 4), and it may be easier to recognize in panel (a) of the new Fig. 7, were we use a linear scale.

   It is true that, with the onset of an ALE, all modes reach a high amplitude more or less simultaneously. We take this as an indication of overlaps between the resonant phase space

islands associated with each harmonic, which means that they are all driven by the same group of energetic ions during the ALE ramp phase. This conjecture remains to be confirmed as pointed out in the "Discussion" section on page 18, lines 2–3.

Although not shown in this paper, we have also performed analyses using Lissajous figures in order to see whether there is some phase locking between different toroidal harmonics before and during an ALE. However, we did not find any clear evidence for such phase locking (see also our reply to comment 5 by Referee 2). Thus, each mode seems to still oscillate at its own pace, even though the amplitudes increase at a similar rate during an ALE.

6. *Fig. 3 and its description — how the mode amplitudes in simulations compare with the experimental results?*

   We believe that we have answered this question in our response to the closely related comment 1 above.

7. *Fig. 5 — description says that the experimental data was decomposed into $n = 1 - 3$ Fourier harmonics. Is there any evidence that the higher n's were not present in the experiments?*

   We have made additional efforts to extract information about $n = 4$, but without success as explained in the new "Methods" section under "Experimental measurements" starting on page 22, line 1. Thus, there is no reliable data for $n > 3$ and this statement was added on page 13, line 19.

8. *It would helpful for the reader, if the descriptions of the results given in the Figure Captions were moved (or duplicated) in the text of the manuscript.*

   We have partially implemented this request and may make further adjustments after consulting with the editors, because there may be additional requests/requirements in this regard in order to comply with the requirement that "Legends should be detailed enough so that each figure and caption can, as far as possible, be understood in isolation from the main text".

We hope that we have been able to adequately answer all questions raised by the referees and would like to thank them once again for their helpful comments.

Reviewers' comments:

Reviewer #1 (Remarks to the Author):

The corrections and additions that the authors have made in response to the review has greatly enhanced the clarity of the paper. This paper is very important and I am happy to recommend that it be published. If the authors could respond to one last thing:
The only comment I have has to do with the discussion section on pg. 16 where it implies that the long-time simulations performed here eliminates the need to tune the input distribution function in order to reproduce the observed amount of transport, however there was not a comparison between simulation and measurements to quantify MHD induced transport presented in the paper. I know that direct comparison of the distribution function to experiment measurements is not possible, but to further support the validation of this simulation, does the simulated distribution function here produce neutron rates consistent with the neutron measurements observed before and after an ALE?

Very nice paper. It was a pleasure to read.

Reviewer #2 (Remarks to the Author):

The authors have made considerable efforts to raise the clarity of their work. In my opinion, this work is appropriate for publication in Nature Communications.

My brief comments on authors' response are as follows.
1. The "1 ms MHD on + 4 ms MHD off" simulations in this work not only demonstrates the possibility of ~100 ms event simulation using the present computing resources, but also shed light on longer time scale gyrokinetic simulations.
2. I agree with the authors that the critical gradient can not be defined in a straightforward way unless specific tools such as transport model is adopted. As a result, while this work can serve as a close-to-first-principle benchmark, simplified models are still important for interpreting and predicting experimental observations.
3. The new discussion is more reasonable than the previous one. The statement in the new discussion about the resonant region filling and its connection to ALE interval can provide the motivation for corresponding analyses and modeling work.
4. The comparison with other approaches is now clarified.
5. I appreciate the authors' clarifying these differences and their thoughts and I agree that understanding the related nonlinear physics is still a subject of ongoing research.

Reviewer #3 (Remarks to the Author):

The revised version of the manuscript is suitable for publication in Nature Communications.

Title:	Simulations tackle abrupt massive migrations of energetic beam ions in a tokamak plasma
Authors:	A. Bierwage, K. Shinohara, Y. Todo, N. Aiba, M. Ishikawa, G. Matsunaga, M. Takechi and M. Yagi

We thank the three referees for carefully checking and evaluating our revised manuscript, and for the very encouraging comments. Below is our answer to the final comment by Referee 1, which is *cited in blue italics*.

Answer to the comment by Referee 1

- *The only comment I have has to do with the discussion section on pg. 16 where it implies that the long-time simulations performed here eliminates the need to tune the input distribution function in order to reproduce the observed amount of transport, however there was not a comparison between simulation and measurements to quantify MHD induced transport presented in the paper. I know that direct comparison of the distribution function to experiment measurements is not possible, but to further support the validation of this simulation, does the simulated distribution function here produce neutron rates consistent with the neutron measurements observed before and after an ALE?.*

  Thank you for this important comment. We forgot to emphasize that the expected neutron rates are consistent with experimental observations and we agree that our discussion can be made more convincing if we emphasize this fact here.

  The required information is already contained in Fig. 11(e) of Ref. 16, where we have scanned the central energetic ion beta value $\beta_{\rm E0}$ and compared the total neutron emission rate with experimental values. That simulation study used the velocity distribution from a conventional orbit-following Monte-Carlo simulation, whose form is similar to the velocity distribution in our present simulations, except for the amplitude, which was used as a free parameter in Ref. 16. The following sentence was added on p. 17 (lines 4–7) of the revised manuscript:

  [...], the energetic ion beta values obtained in the present simulations ($\beta_{\rm E} \approx 1\%$–$1.5\%$ at the centre) imply that the total neutron emission rate of about $(0.6$–$1.0) \times 10^{15}\,{\rm s}^{-1}$ for the N-NB component is consistent with experimental measurements, as was shown in Fig. 11(e) of Ref. 16.

  Note that the total neutron rates found in various experimental papers are higher, sometimes as much as 50%. One obvious reason is that the experiments contain other beams besides the two N-NBs, which is why we write "for the N-NB component" in the newly added sentence. Moreover, one has to keep in mind that the calculated neutron rate strongly depends on the bulk ion density, which is not known accurately for our experiments because $Z_{\rm eff}$ can only be estimated roughly. We expect that the uncertainties are at least 15%, but even this error is difficult to estimate. Due to these difficulties, we usually consider only relative changes in the neutron emission rates; not the absolute values. Nevertheless, we think that the above statement about the *improved consistency* between simulation and experiment is justified.

  Furthermore, note that the total neutron emission rate immediately after an ALE is similar to the emission rate before an ALE (unless the bulk ion density has a steep gradient inside the plasma, which is not typical). The reason is that particle losses are negligible; an ALE merely redistributes the fast ions inside the plasma. The neutron rate drops only gradually after an ALE, and this drop is due to the fact that the particles that are deposited in the cooler periphery slow down more rapidly than those in the hotter core. The variation of the total neutron emission rate between ALEs is only about 10% or even less.

  These points were discussed in Ref. 16, so we have kept the additions made in the present manuscript minimal.

Overleaf, please find p. 17 of the revised manuscript, with the changed portion highlighted in red.

performed here eliminate the need for such tuning by integrating the realistic beam and collision model of the Monte-Carlo simulation into the hybrid code MEGA[25,26]. Absolute quantitative measurements of the energetic ion distribution in ALE experiments on JT-60U are not available, so we are not able to evaluate the accuracy of the new predictions directly. However, the energetic ion beta values obtained in the present simulations ($\beta_{\mathrm{E}} \approx 1\%$–$1.5\%$ at the centre) imply that the total neutron emission rate of about $(0.6–1.0) \times 10^{15}\,\mathrm{s}^{-1}$ for the N-NB component is consistent with experimental measurements, as was shown in Fig. 11(e) of Ref. [16]. Moreover, a recent validation study against a well-diagnosed DIII-D tokamak experiment[27] showed that MEGA results were an order or magnitude more accurate than predictions based on collisional transport only. In that case, the remaining discrepancies fell within experimental error bars of about 20% in the plasma core. The improved predictions for the energetic ion distributions are an essential prerequisite for making reliable predictions for the current drive, torque and heating power that the energetic particle beams exert on the bulk plasma. These factors, in turn, influence many other self-organization processes that occur in magnetically confined plasmas — from microturbulence to large-scale MHD instabilities.

Further work is required in order to fully unravel the mechanisms that trigger ALE-like relaxation events and to become able to construct computationally efficient yet reliable reduced models. The analysis of the simulated ALE dynamics is underway and first new insights were reported here.

We have presented direct evidence, both numerical and experimental, that multiple Alfvén

REVIEWERS' COMMENTS:

Reviewer #1 (Remarks to the Author):

The authors have answered all of my questions. I have no further comment. I recommend publishing this article.
